# Effect of Cinobufacini plus platinum-based chemotherapy regimen on the immune function of patients with non-small-cell lung cancer: A meta-analysis

**DOI:** 10.1016/j.heliyon.2023.e20349

**Published:** 2023-09-20

**Authors:** Yisheng Zhao, Dongwei Zhu, Zhichao Wu, Le Bai, Dan Wang, Yong Xu, Xianmei Zhou

**Affiliations:** a Affiliated Hospital of Nanjing University of Chinese Medicine, Nanjing, 210029, China; b Department of Respiratory and Critical Medicine, Jiangsu Province Hospital of Chinese Medicine, Nanjing, 210029, China; c School of Medicine & Holistic Integrated Medicine, Nanjing University of Chinese Medicine, Nanjing, 210023, China; d School of Chinese Medicine, School of Integrated Chinese and Western Medicine, Nanjing University of Chinese Medicine, Nanjing, 210023, China

**Keywords:** Cinobufacini, NSCLC, Platinum-based chemotherapy, Immune function, Meta-analysis

## Abstract

**Background:**

Cinobufacini is a Chinese medicinal preparation extracted from the traditional Chinese medicine toad skin and is commonly used clinically as an adjuvant treatment for malignant tumours.

**Purpose:**

To systematically evaluate the effects of Cinobufacini combined with a first-line platinum-based chemotherapy regimen in patients with non-small-cell lung cancer (NSCLC), especially in terms of immune function.

**Materials and methods:**

Eight electronic databases were searched for randomised controlled trials (RCTs) investigating Cinobufacini in conjunction with platinum-based chemotherapy for NSCLC (stage III-IV) published from 2012 to the present. GRADE Pro GDT was used to assess RCT quality and meta-analysis was performed mainly using Review Manager version 5.4, with the assistance of Stata version 16.0 (StataCorp LLC, College Station, TX, USA), and trial sequential analysis software.

**Results:**

A total of 35 studies were included. Meta-analysis revealed that the combination therapy group exhibited a better disease control rate (DCR) [OR = 2.63, 95%CI (2.15, 3.21), *P* < 0.00001], with a higher one-year [OR = 2.41,95% CI (1.75,3.33), *P* < 0.00001], and two-year [OR = 2.28, 95% CI (1.56,3.33), *P* < 0.00001] survival rate, plus lower leukocyte toxicity [OR = 0.40, 95%CI (0.33,0.49), *P* < 0.00001]. For immune function, the combination of chemotherapy with Cinobufacini effectively increased the proportion of CD3^+^ [SMD = 1.15, 95% CI (0.89,1.42), *P* < 0.00001], CD4^+^ [SMD = 1.60, 95%CI (1.26,1.94), *P* < 0.00001] and the CD4^+^/CD8^+^ ratio [SMD = 2.15, 95% CI (1.45,2.86), *P* < 0.00001] in peripheral blood.

**Conclusion:**

The addition of Cinobufacini to platinum-based chemotherapies for advanced NSCLC significantly improved clinical efficacy, enhanced immune function, and reduced chemotherapeutic toxicity, irrespective of administration and treatment duration.

## 1 Introduction

Of all cancers, lung cancer ranks second in morbidity and remains the leading cause of cancer-related deaths [1], accounting for 11.4% of all malignancies and 18% of all cancer deaths [2]. Globally, approximately 2 million individuals are diagnosed with lung cancer each year, and approximately 1.76 million die from the disease [3]. Approximately 85% are diagnosed with non-small-cell lung cancers (NSCLCs) [4], with five-year survival rates ranging from 14% to 49% for stage I to IIIa NSCLCs. In comparison, the five-year survival rate for stage IIIb/IV NSCLC is reported to be <5% [5]. Currently, two-drug platinum-containing chemotherapy regimens are the first-line drug treatments for advanced NSCLC. Although chemotherapy can inhibit tumour growth and effectively reduce tumour size, it can also lead to many serious toxic side effects, one of the most frequent of which is bone marrow suppression [6]. Therefore, many studies are investigating ways to enhance the effects of chemotherapy while minimising toxic side effects.

In recent years, Chinese medicine has been applied as an adjuvant to the treatment of many malignant tumours, including lung cancer, and has achieved good therapeutic effects and considerably improved patient survival quality. Cinobufacini is an extract of toad skin, a traditional Chinese medicine derived from the outer skin of *Bufo Gargarizans* and *Bufo Melanostictus Schneider* [7], and is obtained by hydro extraction and alcoholic sedimentation [8]. Its main active ingredients with anticancer effects include toad dihydroxylation lactones, alkaloids, and peptides such as toad venom, toad thiazide, and cyclic dipeptide [9]. It is widely used as an adjuvant treatment of colorectal, lung, liver, oesophageal, and gastric cancers, and other malignant tumours [10]. Several clinical trials have reported that it can inhibit tumour growth, reduce tumour volume, lower the level of tumour markers and the incidence of toxic reactions to a certain extent, and improve the quality of life of patients, which helps prolong the survival of those with advanced malignant tumours [11].

**Fig. 1:**
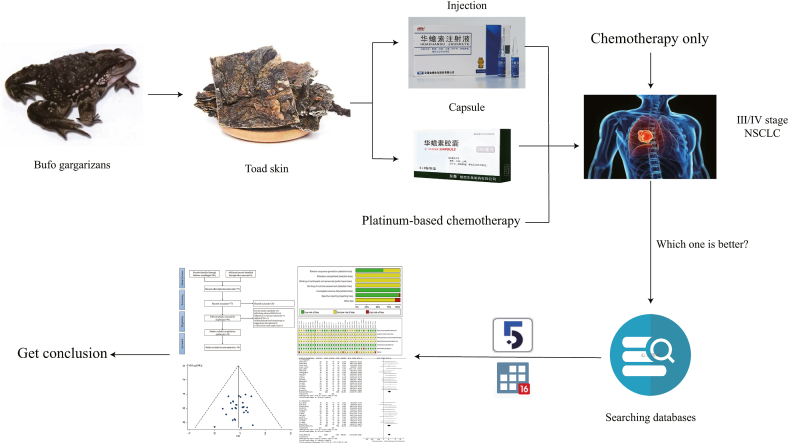
Workflow of the present study.

Many randomised controlled trials (RCTs) have reported that Cinobufacini, as an adjuvant agent in combination with platinum-based chemotherapy, can increase efficacy and reduce toxicity in the treatment of advanced NSCLC. Some studies have also evaluated the overall efficacy and safety of Cinobufacini in NSCLC [12,13], but none have systematically analyzed its effect on immune function. As such, the present investigation aimed to collect RCTs investigating the effect of different formulations of Cinobufacini on immune function in conjunction with platinum-based chemotherapy regimens for NSCLC over the past 10 years and to perform a comprehensive systematic evaluation using meta-analysis to provide an evidence-based medical rationale for its clinical application. The general flow of the study is illustrated in Fig. 1.

## 2 Materials and methods

This meta-analysis was performed in accordance with the Preferred Reporting Items for Systematic Reviews and Meta-Analyses (i.e., “PRISMA”) criteria^S1^ (#CRD 42022350367).

### 2.1 Literature search

Eight electronic databases were searched for relevant studies: Chinese National Knowledge Infrastructure (CNKI), WanFang, VIP, China Biological Medicine Database (CBM), PubMed, Embase, The Cochrane Library, and Web of Science. The literature search was designed to retrieve all relevant studies published between 2012 and July 1, 2023. A combination of the following terms was used to search the English databases: Cinobufacini AND (chemotherapy OR chemotherapy) AND (non-small-cell lung cancer [mesh] OR non-small-cell lung carcinoma [mesh] OR carcinoma, non-small-cell lung [mesh] OR Lung Carcinoma, non-small-cell [mesh] OR NSCLC). The following search terms were used for the Chinese databases: [Huachansu] AND [Feixiaoxibao feiai] AND [Hua liao].

### 2.2 Inclusion criteria

All included studies included were determined to be RCTs, with patients not restricted by sex, race, or age. The study subjects fulfilled the criteria of the 2021 edition of the Chinese Society of Clinical Oncology (CSCO) Guidelines for the Management of Primary Lung Cancer and were cytologically or histopathologically diagnosed with advanced NSCLC (clinical stage III-IV). The trial group was treated with Cinobufacini plus platinum-based chemotherapy meanwhile the control group was treated with a platinum-based chemotherapy regimen(s) only, both with unlimited dose and duration of treatment.

### 2.3 Exclusion criteria

Duplicate studies, and those with inconsistent baseline information regarding different groups, investigations with incomplete data or inaccessible full text, unknown interventions, unclear description of the treatment course, unclear description of the efficacy evaluation criteria or statistical errors, were excluded.

### 2.4 Outcome measures

Immune function and survival rate were the primary outcomes, and recent clinical efficacy and adverse effects were the secondary endpoints. Studies investigating at least one of the above indicators were also included. Efficacy rate was determined according to the World Health Organization (WHO) criteria for solid tumours as complete remission (CR), partial remission (PR), stabilisation of disease (SD), and progression of disease (PD), with disease control rate (DCR) calculated as= (number of CR cases + number of PR cases + number of SD cases)/total cases. The long-term clinical efficacy outcomes included one- and two-year survival rates. Immune function indicators included T-lymphocyte subsets (CD3^+^, CD4^+^, and CD4^+^/CD8^+^) and natural killer (NK) cells in the peripheral blood. Adverse reactions, specifically leukocyte toxicity, were evaluated according to the Common Terminology Criteria for Adverse Events (CTCAE) version 4.0.

### 2.5 Data extraction

Data were extracted independently by two reviewers according to data extraction requirements, which included the basic characteristics of the included studies (first author, title of the study, year of publication), basic patient information (sex, age), interventions and controls (control drugs, dose of medication, frequency of medication), outcome indicators, and evaluation of study quality. Any disputes(s) during data extraction was resolved by a third reviewer.

### 2.6 Quality assessment

The quality of the included studies was independently evaluated by two researchers based on the Cochrane Handbook's Risk of Bias Assessment Tool in the following areas: generation of random sequences; concealment of random assignment; blinding of patients, investigators, and outcome evaluators; completeness of outcome data; selective reporting of study results; and other biases. If any disagreement was encountered, a third reviewer discussed and resolved the issue.

### 2.7 Statistical analysis

Review Manager version 5.4 provided by the Cochrane Collaboration Network and Stata version 16.0 (StataCorp LLC, College Station, TX, USA) were used to perform the meta-analyses with a test level of α = 0.05. Odds ratio (OR) was used for binary variables and standardized mean difference (SMD) was used for continuous variables. This study used the I^2^ statistic to test for heterogeneity among the included studies. An I^2^ ≥ 50% was regarded as evidence of substantial heterogeneity, and a random-effects model was applied, whereas an I^2^ < 50%, indicated low heterogeneity, and a fixed-effects model was applied. Potential publication bias was analyzed using funnel plots for meta-analyses of ≥10 studies, and Egger's test was performed to assess asymmetry in the funnel plot. Subgroup analysis was used to test for heterogeneity, and the impact of several factors on the effect size was examined. The stability of the results was further assessed using the sensitivity analysis function in Stata version 16.0. Trial sequential analysis (TSA) software was used to assess the robustness of the results and to calculate the required information size (RIS) to draw conclusions. Meta-regression analysis was used to test the effects of other factors on the results.

## 3 Results

### 3.1 Literature search and study characteristics

**Fig. 2:**
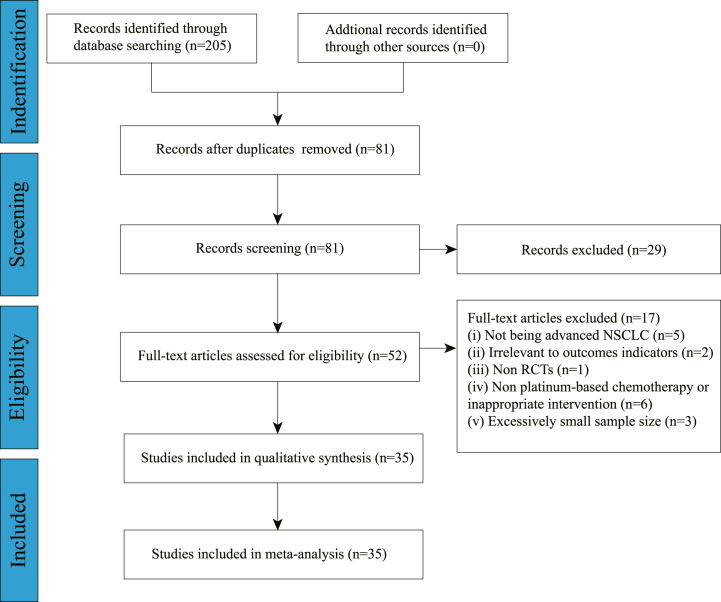
PRISMA search diagram.

As indicated in Fig. 2, the literature search retrieved a total of 205 studies (CNKI: 82, WanFang: 61, VIP: 39, CBM: 23, PubMed: 0, EMBASE: 0, Cochrane Library: 0, Web of Science: 0), 35 of which were ultimately included [[14], [15], [16], [17], [18], [19], [20], [21], [22], [23], [24], [25], [26], [27], [28], [29], [30], [31], [32], [33], [34], [35], [36], [37], [38], [39], [40], [41], [42], [43], [44], [45], [46], [47], [48]], with 2967 patients.

As presented in Table 1, authors, along with the year of publication, sample size, TNM stage, Cinobufacini formulation, dosage and administration of Cinobufacini, treatment duration, and outcomes were all baseline characteristics of the included studies. All of the included studies were conducted in China; among these, 20 used capsules and 15 used injections.

**Table 1:** Baseline characteristics of included studies.

Study	Cases	Stage	Formulation	Intervention		Dosage and administration	Duration（day）	Outcome
	T/C			T	C			
Wei and Xu., 2017	34/34	Ⅲ-Ⅳ	Capsule	DP + Cinobufacini	DP	0.5 g·tid·po	28	①⑧
Huang, 2019	43/43	Ⅲb-Ⅳ	Capsule	GP + Cinobufacini	GP	0.5 g·bid·po	42	①④⑤⑥⑦
Liu, 2018	40/40	Ⅲb-Ⅳ	Capsule	TP,NP + Cinobufacini	TP,NP	0.6 g·bid·po	42	①②⑧
Li. 2019	38/37	Ⅲ-Ⅳ	Capsule	DP + Cinobufacini	DP	0.5 g·tid·po	42	①④⑤⑥
Chen et al., 2019	36/31	Ⅲ-Ⅳ	Capsule	DP + Cinobufacini	DP	0.5 g·tid·po	42	①②③④⑤⑥⑧
Chen et al., 2016	40/40	Ⅲb-Ⅳ	Capsule	GP + Cinobufacini	GP	0.6 g·bid·po	42	①②③⑤⑥
Hao et al., 2019	44/44	Ⅲ-Ⅳ	Capsule	GP + Cinobufacini	GP	0.5 g·bid·po	42	①⑤⑥
Su, 2019	44/44	Ⅲb-Ⅳ	Capsule	GP + Cinobufacini	GP	0.6 g·bid·po	84	①⑧
Pu et al., 2017	42/38	Ⅲ-Ⅳ	Capsule	NP + Cinobufacini	NP	0.5 g·bid·po	56	①②③④⑤⑥⑦
Lan et al., 2018	43/42	Ⅲ-Ⅳ	Capsule	PC + Cinobufacini	TC	0.5 g·tid/qid·po	63	①③
Chen et al., 2017	36/36	Ⅲb-Ⅳ	Capsule	PC + Cinobufacini	TC	0.6 g·bid·po	84	①④⑤⑥
Guan et al., 2021	36/35	Ⅲb-Ⅳ	Capsule	TP + Cinobufacini	TP	0.5 g·tid/qid·po	126	①④⑤⑥
Miao et al., 2014	30/30	Ⅲ-Ⅳ	Capsule	TP + Cinobufacini	TP	0.5 g·tid·po	56	①⑧
Chen et al., 2018	31/31	Ⅲ-Ⅳ	Capsule	AP,GP + Cinobufacini	AP,GP	0.5 g·bid·po	42	①④⑤⑥
Li et al., 2018	58/58	Ⅲb-Ⅳ	Capsule	GP + Cinobufacini	GP	0.75 g·tid·po	56	①③④⑤⑥⑧
Liu, 2014	45/40	Ⅲa-Ⅳ	Capsule	NP,TP + Cinobufacini	NP,TP	0.5 g·bid·po	42	①②③⑧
Yu et al., 2018	33/30	Ⅲ-Ⅳ	Capsule	PC,GP + Cinobufacini	AP,GP	0.5 g·tid·po	42	①⑧
Zhao, 2021	29/29	Ⅲ-Ⅳ	Capsule	GP + Cinobufacini	GP	0.75 g·tid·po	56	①④⑤⑥⑧
Li, 2023	47/47	Ⅲ-Ⅳ	Capsule	AP + Cinobufacini	AP	0.5 g·tid·po	42	①②③④⑤
Sun et al., 2022	49/48	Ⅲ-Ⅳ	Capsule	GP + Cinobufacini	GP	0.75 g·tid·po	42	①④⑤⑥⑧
Chi et al., 2019	49/49	Ⅲb-Ⅳ	Injection	DC + Cinobufacini	DC	10–20 ml·qd·ivgtt	84	①⑧
Yu et al., 2012	32/32	Ⅲ-Ⅳ	Injection	DP + Cinobufacini	DP	20 ml·qd·ivgtt	28	①
Lu et al., 2015	31/31	Ⅲa-Ⅳ	Injection	NP + Cinobufacini	NP	20 ml·qd·ivgtt	20	①
Wang et al., 2013	45/45	Ⅲb-Ⅳ	Injection	TP + Cinobufacini	TP	20 ml·qd·ivgtt	42	①
Lan, 2017	48/48	Ⅲ-Ⅳ	Injection	TP + Cinobufacini	TP	20 ml·qd·ivgtt	28	①⑧
Yao et al., 2018	100/100	Ⅲ-Ⅳ	Injection	DP + Cinobufacini	DP	20 ml·qd·ivgtt	15	②④⑤⑥⑧
Yin et al., 2018	60/60	Ⅲb-Ⅳ	Injection	EP + Cinobufacini	EP	20 ml·qd·ivgtt	84	①
Bian et al., 2015	32/31	Ⅲ-Ⅳ	Injection	GP + Cinobufacini	GP	20 ml·qd·ivgtt	84–126	①
Hu, 2012	36/38	Ⅲb-Ⅳ	Injection	TP + Cinobufacini	TP	20 ml·qd·ivgtt	56–84	①②③⑧
Ji et al., 2017	49/49	Ⅲb-Ⅳ	Injection	DC + Cinobufacini	DC	20 ml·qd·ivgtt	56	①⑧
Cao et al., 2016	40/40	Ⅳ	Injection	DP + Cinobufacini	DP	10–20 ml·qd·ivgtt	84	①⑧
Dong, 2013	46/40	Ⅳ	Injection	AP + Cinobufacini	AP	30 ml·qd·ivgtt	60	①④⑤⑥⑧
Zhou, 2014	47/47	Ⅲb-Ⅳ	Injection	TP + Cinobufacini	TP	20 ml·qd·ivgtt	42	①
Chen, 2016	45/45	Ⅲ-Ⅳ	Injection	GP + Cinobufacini	GP	20 ml·qd·ivgtt	45	①②③⑧
Wang et al., 2018	38/39	Ⅲ-Ⅳ	Injection	TP + Cinobufacini	TP	20 ml·qd·ivgtt	56	①②④⑤⑥⑦⑧

Note. (1) T is the test group; C is the control group; AP: pemetrexed + cisplatin; DP: docetaxel + cisplatin; GP: gemcitabine + cisplatin; NP: vinorelbine + cisplatin; TC: paclitaxel + carboplatin; TP: paclitaxel + cisplatin; EP: Etoposide + cisplatin; (2)Outcome index:①DCR; ②One-year survival rate; ③Two-year survival rate; ④CD_3_^+^;⑤CD_4_^+^;⑥CD_4_^+^/CD_8_^+^;⑦NK; ⑧Leukocyte toxicity.

### 3.2 Evaluation of methodological quality

**Fig. 3:**
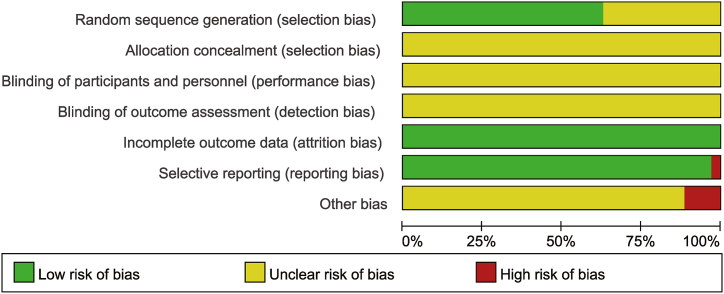
Risk of bias graph.

**Fig. 4:**
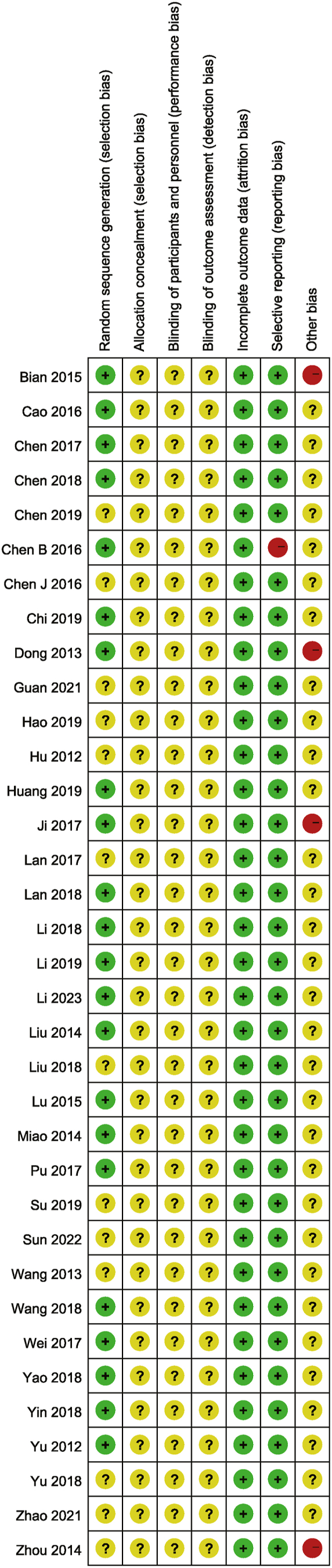
Risk of bias summary.

As shown in Fig. 3, Fig. 4, of all studies, 22 were rated as “low risk” due to the use of randomised numerical tables, 13 mentioned randomisation without defining the methodology and were rated as “unclear”, 35 were rated as “uncertain” because it was unclear whether allocation concealment was applied, 35 were classified as “unclear” because they could not be verified whether blinding was used, no study mentioned dislodged subjects and was rated as “low risk”, one study with immune function reporting only histograms without labeling or reporting the corresponding raw data, which could not be extracted, was rated “high risk”. The other 34 studies were rated as “low risk” because they did not identify missing data, two studies reported inconsistent patient numbers and were rated as “high risk”, two studies reported similar data and were rated as “high risk”, and in the remaining 31 studies, it could not be determined whether other biases existed and were rated as “unclear”.

### 3.3 Outcome measures

#### 3.3.1 DCR

**Fig. 5:**
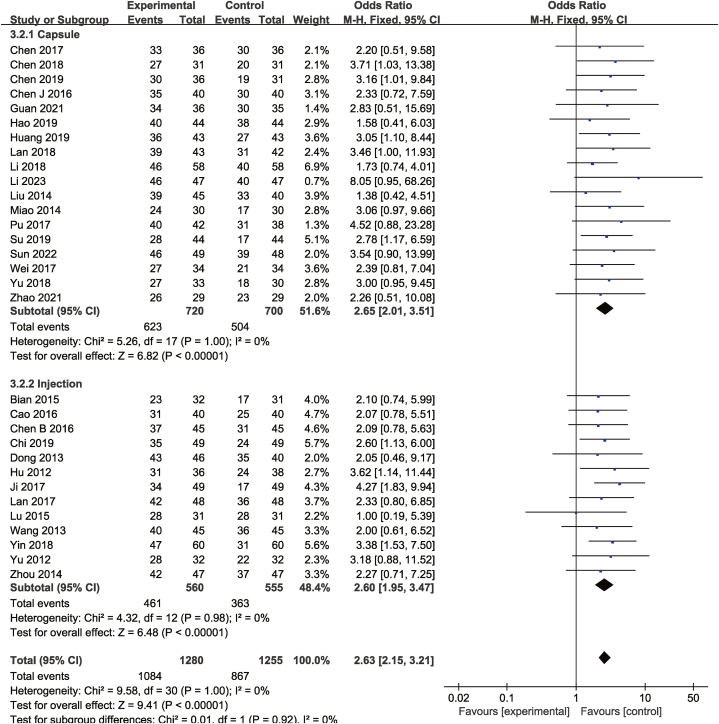
Meta-analysis of DCR.

Thirty-one studies compared DCRs between the test and control groups. Assuming statistical homogeneity to be present (*P* = 1.00, I^2^ = 0%), a fixed-effects model was used. As presented in Fig. 5, compared with chemotherapy alone, the combination of Cinobufacini and chemotherapy was more successful in increasing DCR [OR = 2.63, 95%CI (2.15, 3.21), *P* < 0.00001].

#### 3.3.2 Survival rate

**Fig. 6:**
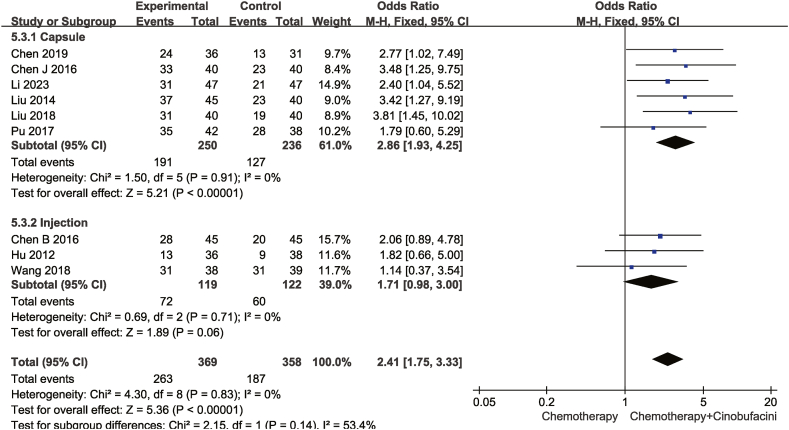
Meta-analysis of one-year survival rate.

**Fig. 7:**
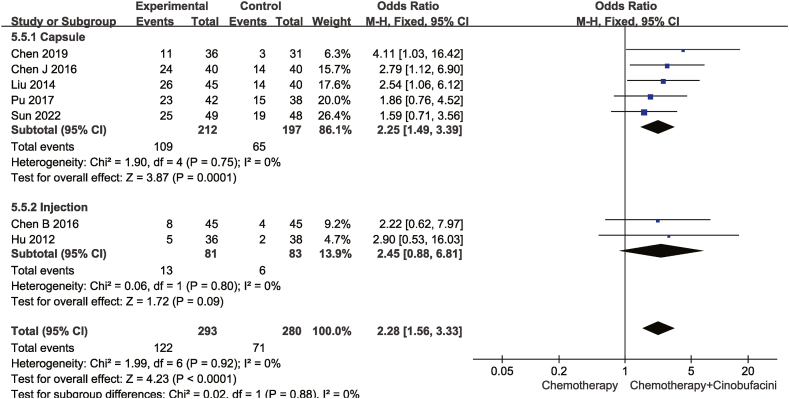
Meta-analysis of two-year survival rate.

Nine studies compared one-year survival rate, whereas seven compared two-year survival rate between the test and control groups. As shown in Fig. 6, Fig. 7, the heterogeneity test revealed no significant heterogeneity. Consequently, a fixed-effects model was adopted. The combination therapy group exhibited a higher one-year [OR = 2.41,95% CI (1.75,3.33), *P* < 0.00001], and two-year [OR = 2.28, 95% CI (1.56,3.33), *P* < 0.00001] survival rate.

#### 3.3.3 Immune function

**Fig. 8:**
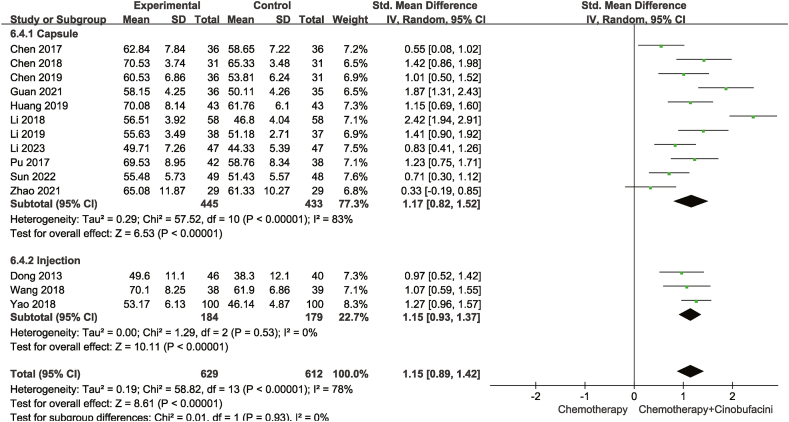
Meta-analysis of CD3^+^.

**Fig. 9:**
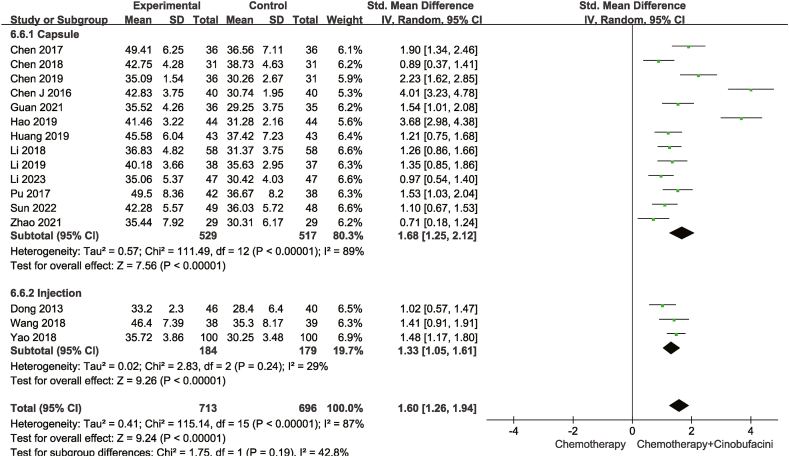
Meta-analysis of CD4^+^.

**Fig. 10:**
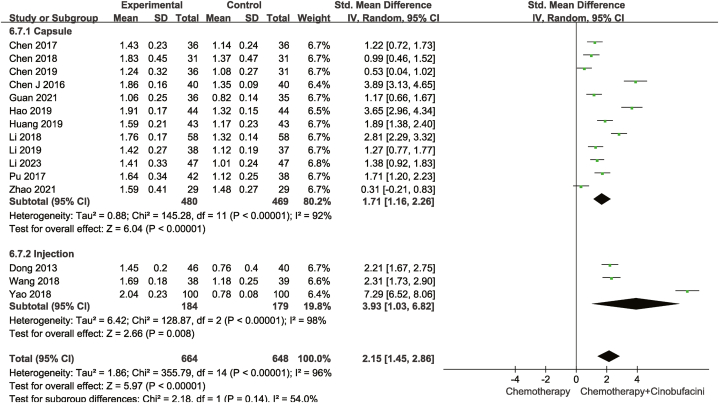
Meta-analysis of CD4^+^/CD8^+^.

Fourteen studies compared CD3^+^ levels, whereas sixteen compared CD4^+^ and fifteen CD4^+^/CD8^+^ levels in the peripheral blood between the test and control groups. As described in Fig. 8, Fig. 9, Fig. 10, according to the heterogeneity test results, a random-effects model was adopted for statistical analyses. The combination of chemotherapy with Cinobufacini effectively increased the proportion of CD3^+^ [SMD = 1.15, 95% CI (0.89,1.42), *P* < 0.00001], CD4^+^ [SMD = 1.60, 95%CI (1.26,1.94), *P* < 0.00001] cells and CD4^+^/CD8^+^ ratio [SMD = 2.15, 95% CI (1.45,2.86), *P* < 0.00001] in the peripheral blood.

As shown in Table 2, three studies^15, 22, 46^ compared NK cell levels between the two groups after treatment. Results revealed that the combined treatment was significantly superior to the control.

**Table 2:** Comparison of NK%.

	T (pre.)	C (pre.)	*P*	T (post.)	C (post.)	*P*
Huang, 2019	13.86 ± 3.29	14.08 ± 3.34	＞0.05	20.53 ± 4.08	13.74 ± 4.19	＜0.05
Pu et al., 2017	21.48 ± 7.35	20.89 ± 6.83	＞0.05	24.67 ± 7.05	18.96 ± 5.04	＜0.05
Wang et al., 2018	13.60 ± 4.52	14.10 ± 5.77	＞0.05	21.2 ± 3.86	13.8 ± 4.28	＜0.05

T is the test group; C is the control group.

#### 3.3.4 Leukocyte toxicity

**Fig. 11:**
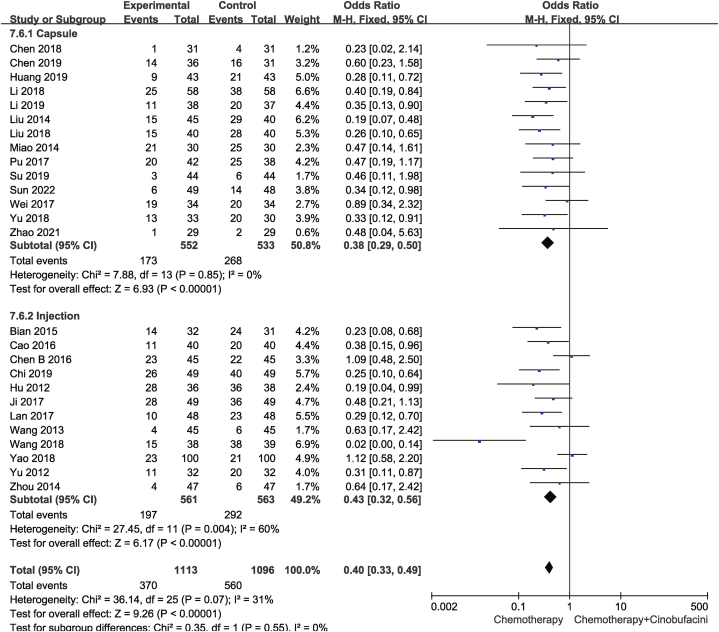
Meta-analysis of leukocyte toxicity.

Twenty-six studies compared the incidence of leukocyte count reduction between the test and control groups. The fixed-effects model was used given statistical homogeneity (*P* = 0.07 and I^2^ = 31%). As shown in Fig. 11, chemotherapy combined with Cinobufacini was effective in lowering the incidence of leukopenia [OR = 0.40, 95%CI (0.33,0.49), *P* < 0.00001].

#### 3.3.5 Subgroup and sensitivity analyses

In this study, high heterogeneity was noted among the three included outcomes for CD3^+^ (I^2^ = 78%), CD4^+^ (I^2^ = 87%) and CD4^+^/CD8^+^ (I^2^ = 96%), whereas there was acceptable homogeneity among the included studies for the remaining outcomes.

We performed subgroup analyses of all outcomes based on formulation and subgroup analyses of outcomes for no less than eight studies included based on the duration of administration of Cinobufacini, and examined the effect of individual studies on the total combined effect size in the above three outcomes with high heterogeneity using Stata version 16.0.

As shown from Fig. 5, Fig. 6, Fig. 7, Fig. 8, Fig. 9, Fig. 10, Fig. 11, capsules were more effective than injections in terms of one-year survival rate. The results for the remaining outcomes did not differ significantly between capsules and injections. As presented in Table 3, a dosing duration of 15–42 days resulted in a higher one-year survival rate, whereas the remainder of the results were not significantly related to the duration of dosing. However, neither of the above differences was statistically significant (*P* ≥ 0.05), suggesting that they were both not the source of heterogeneity.

**Table 3:** Subgroup analysis according to durations of Cinobufacini.

Subgroups	DCR OR (95%CI)	One-year survival rate OR (95%CI)	Leukocyte toxicity OR (95%CI)	CD_3_^+^ SMD(95%CI)	I^2^	CD_4_^+^ SMD(95%CI)	I^2^	CD_4_^+^/CD_8_^+^ SMD(95%CI)	I^2^
15 d-42 d	2.53 (1.85,3.46)	3.08 (2.01,4.71)	0.43 (0.33,0.56)	1.10 (0.90, 1.30)	33%	1.83 (1.26, 2.41)	92%	2.59 (1.31, 3.86)	98%
43 d-126 d	2.70 (2.08,3.52)	1.73 (1.05,2.84)	0.37 (0.28,0.49)	1.20 (0.67, 1.73)	88%	1.33 (1.06, 1.59)	52%	1.67 (1.04, 2.30)	90%
*p* between subgroups	0.75	0.08	0.42	0.73		0.12		0.21	

**Fig. 12:**
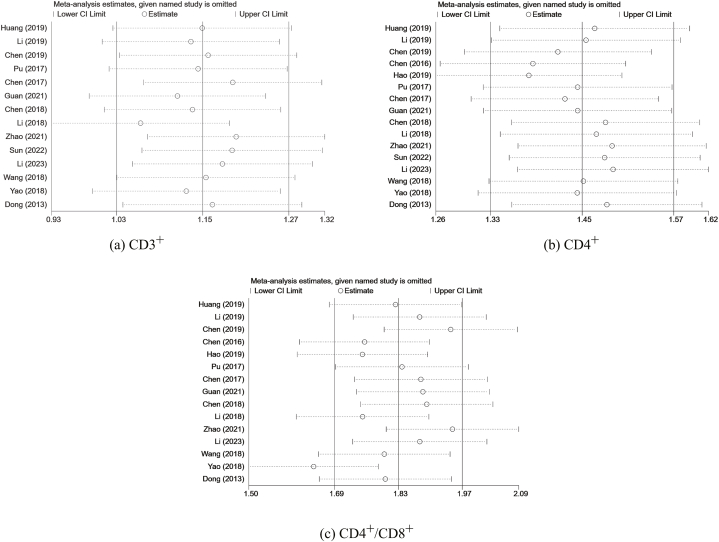
Sensitivity analyses (a)CD3^+^ (b)CD4^+^ (c)CD4^+^/CD8^+^.

The pooled SMD of CD3^+^and CD4^+^ did not significantly change, as shown in Fig. 12(a and b). However, the pooled SMD of CD4^+^/CD8^+^ [Fig. 12(c)] changed significantly after excluding the study codenamed Yao 2018, implying that it was the main source of heterogeneity. We scrutinized this study but did not find sufficient evidence to rule it out.

All the 95% CI did not cross the invalid line of “0”, suggesting good stability of the results.

#### 3.3.6 Publication bias

**Fig. 13:**
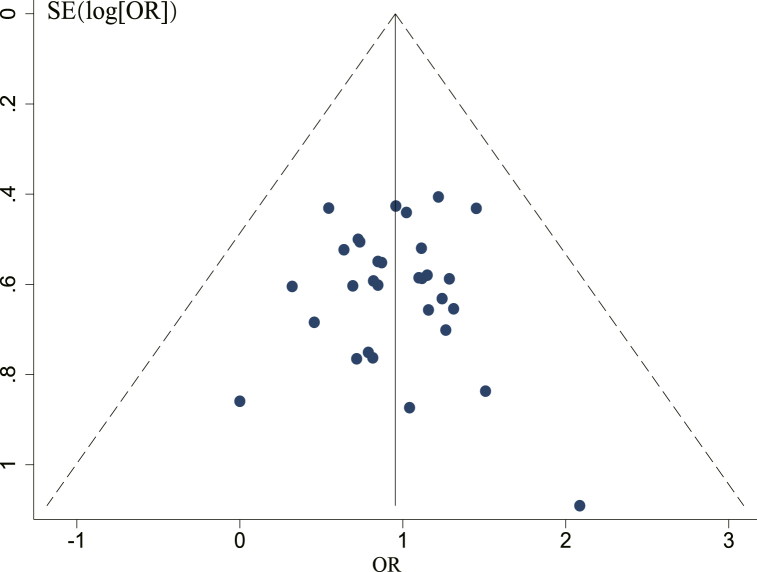
Funnel plot of DCR.

**Fig. 14:**
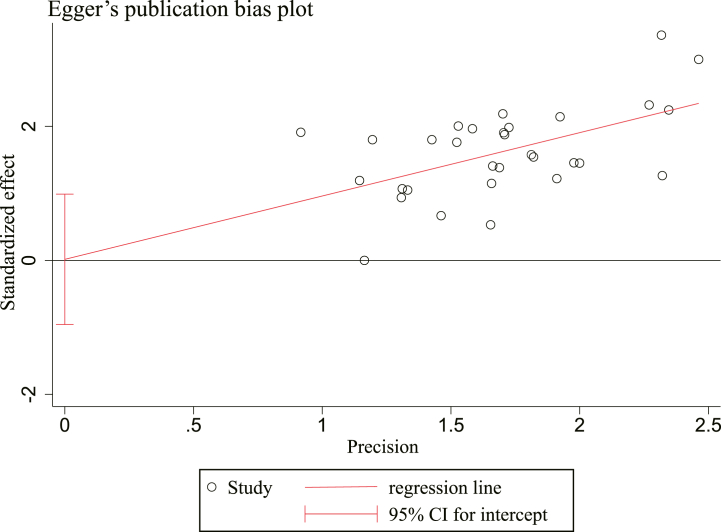
Egger's publication bias plots of DCR.

Publication bias in studies reporting DCR was assessed using funnel plots and Egger's tests. A possible publication bias is indicated by small asymmetry between the left and right sides of the funnel plot (Fig. 13). Consequently, a quantitative analysis using Egger's test (Fig. 14) was performed, and the outcomes [t = 0.03, 95% CI (−0.9581169,0.9889536), *P* = 0.974] demonstrated no proof of publication bias.

#### 3.3.7 TSA and meta-regression analysis

**Fig. 15:**
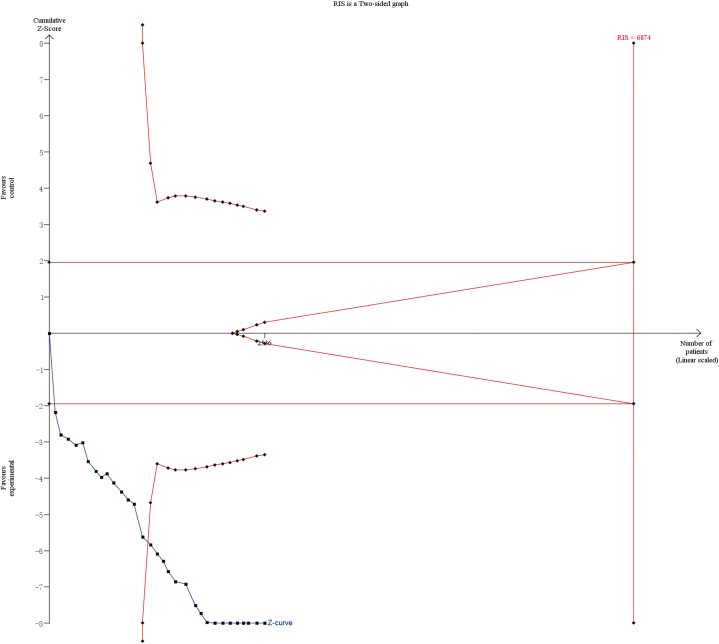
Trial sequential analysis of the DCR.

TSA was performed to reduce false-positive results owing to random errors. TSA (Fig. 15) revealed that the Z curve for DCR intersected with the conventional boundary and TSA boundary but not with the required information size (RIS) line, indicating that the results of this meta-analysis are still robust even if RIS is not reached.

**Fig. 16:**
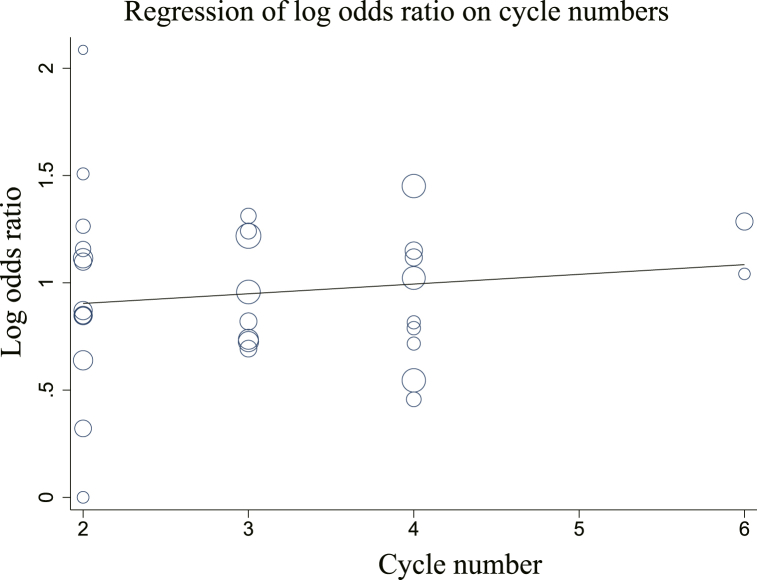
Meta-regression analysis of DCR.

Meta-regression analysis (Fig. 16) demonstrated no significant linear relationship between DCR and the number of treatment cycles; however, there was a tendency slight increase with an increase in the number of cycles (log OR = 2.255 + 1.046 cycles, *u* = 0.44, *P* = 0.661).

#### 3.3.8 Quality of evidence

Two reviewers used GRADE Pro GDT to assess the quality of evidence for each outcome in terms of risk of bias, inconsistency, indirectness, imprecision, and publication bias. The following four categories of evidence quality were determined and assigned: very low, low, moderate, and high. All evidence was of high quality by default before the evaluation. All of the included studies had a few deficiencies in randomisation, allocation concealment, and blinding, with reduced levels of risk of bias. In addition, the results for CD3^+,^ CD4^+^, and CD4^+^/CD8^+^ cells were heterogeneous. Therefore, their quality grades were reduced using a single scale. The two-year survival rate was reduced in terms of precision owing to the small number of included cases (≤300 cases). The final results are summarized in Table 4, with three outcomes of moderate quality and four of low quality.

**Table 4:** GRADE evidence profile.

Outcomes	Quality assessment					No. Of patients		Clinical efficacy and safety		Quality
	risk of bias	Inconsistency	Indirectness	Imprecision	Publication bias	Trails	Control	Odds ratio (95% CI)	Absolute (95% CI)	
GRADE evidence profile of efficacy outcomes										
DCR (31)	Serious^1^	No	No	No	None	1084/1280 (84.7%)	867/1255 (69.1%)	OR 2.63 (2.15–3.21)	164 more per 1000 (from 137 more to 187 more)	⊕⊕⊕○ MODERATE
One-year survival rate (9)	Serious^1^	No	No	No	None	263/369 (71.3%)	187/358 (52.2%)	OR 2.41 (1.75–3.33)	203 more per 1000 (from 134 more to 262 more)	⊕⊕⊕○ MODERATE
Two-year survival rate (7)	Serious^1^	No	No	Serious^3^	None	122/293 (41.6%)	71/280 (25.4%)	OR 2.28 (1.56–3.33)	183 more per 1000 (from 93 more to 277 more)	⊕⊕○○○○ LOW
GRADE evidence profile of immune outcomes										
Leukocyte toxicity (26)	Serious^1^	No	No	No	None	370/1113 (33.2%)	560/1096 (51.1%)	OR 0.40 (0.33–0.49)	216 fewer per 1000 (from 172 fewer to 255 fewer)	⊕⊕⊕○ MODERATE
CD_3_^+^(14)	Serious^1^	Serious^2^	No	No	None	629	612	No	SMD 1.15 higher (0.89–1.42 higher)	⊕⊕○○○○ LOW
CD_4_^+^(16)	Serious^1^	Serious^2^	No	No	None	713	696	No	SMD 1.6 higher (1.26–1.94 higher)	⊕⊕○○○○ LOW
CD_4_^+^/CD_8_^+^(15)	Serious^1^	Serious^2^	No	No	None	664	648	No	SMD 2.15 higher (1.45–2.86 higher)	⊕⊕○○○○ LOW

DCR, disease control rate; OR, odds ratio; CI, confidence intervals; WMD, weighted mean difference; CD, cluster of differentiation.

1 The included studies have certain defects or risks in randomisation, allocation concealment, and blinding.

2 The included studies are highly or moderately heterogeneous.

3 The sample size for each group of the outcome is fewer than 300 cases, which reduces the robustness of these results.

## 4 Discussion

The current study included 35 RCTs with a total of 2967 patients. The results revealed that the combination of Cinobufacini and chemotherapy effectively enhanced immune function, improved efficacy, and reduced leukocyte toxicity.

DCR is one of the main indicators for evaluating recent clinical efficacy, while one-year and two-year survival rates directly reflect the survival benefit to patients and are considered to be the gold standards for long-term efficacy [12]. Results of the present meta-analysis revealed that Cinobufacini combined with chemotherapy performed well in improving these important indicators and had definite efficacy in NSCLC. Meta-regression analysis revealed that, although DCR tended to rise with the number of treatment cycles, given the small number of studies with six cycles and *P* ≥ 0.05, there was no supportive evidence to conclude that DCR would increase with an increase in cycles.

The total number of leukocytes can reflect immune function. Therefore, we selected leukocyte toxicity as an observational indicator to evaluate changes in immune function, and the results demonstrated that both formulations of Cinobufacini combined with chemotherapy reduced the incidence of leukocyte toxicity in patients. Peripheral blood lymphocyte levels are significantly lower in patients with lung cancer than in normal subjects and further decrease with the progression of the disease and the intervention of cytotoxic treatments such as chemotherapy [49]. Cellular immunity is critical for suppressing tumour development and progression, in which T cell-mediated anti-tumour immune effects play an important role. Its monitoring of tumour escape is achieved by surveillance and killing of tumour cells in the organism [50]. Some clinical studies have shown that the number and function of T-lymphocyte subpopulations in advanced lung cancer patients are positively correlated with patient survival, radiotherapy efficacy and quality of life [51,52]. We focused on T-lymphocyte subsets in the peripheral blood (CD3^+^, CD4^+^ and CD4^+^/CD8^+^) as representative indicators of immune function. CD3^+^ is present only on the surface of T cells and consists mainly of six peptide chains, which function to transduce the activation signals generated by the recognition of antigens by the T cell antigen receptor T cell receptor (TCR) [53]. Peripheral blood CD3^+^ levels reflect the overall status of the immune cells. CD4^+^ T cells, as a type of helper T lymphocytes, recognise soluble antigens secreted by tumour cells and participate in the activation of B cells, cytotoxic T cells, macrophages and NK cells, thus, increasing and expanding the immune response. They release a variety of cytokines such as IL-2, INF-γ, and TNF-β to participate in anti-tumour effects directly [54]. They play a coordinated role in the immune response of the whole body, thereby inhibiting the growth and metastasis of the tumour [55]. The CD4^+^/CD8^+^ ratio indicates the positive regulation state of cellular immune balance, and its decrease indicates weakness of cellular immunity [56]. Its continuous decreasing trend and lower ratio are high-risk factors for the recurrence and metastasis of lung cancer [57]. NK cells are the main cells that mediate the immune response by secreting cytokines such as IFN-α, IFN-γ, IL-2, and GM-CSF, and kill tumour cells non-specifically [58]. During the immunological escape of cancer cells, both their function and number are significantly reduced [59]. Meta-analysis revealed that both doses of Cinobufacini combined with chemotherapy increased the proportion of CD3^+^ and CD4^+^ in the peripheral blood of patients and increased the CD4^+^/CD8^+^ ratio, which was statistically different compared with chemotherapy alone. The number of NK cells also increased after combination therapy. Some studies have reported that the active components of Cinobufacini, such as ester toad venom ligands, can increase the secretion of immune-related cytokines in tumour-bearing nude mice, enhance the expression of T-helper (Th) 1- and Th2-related cytokines, promote cellular and humoral immunity, and enhance immune function in the body [60], which corresponds to the results of the present study, indicating that Cinobufacini has a positive regulatory effect on immune function.

In the present study, we performed a subgroup analysis of two different formulations of Cinobufacini. Both capsules and injection solutions were made from dried toad skin extract and had the same active ingredients. Although there was no statistical difference, the results showed that oral administration was effective in improving long-term survival, whereas intravenous administration was largely influenced by patient compliance and the studies included were insufficient, so an accurate conclusion about which of the two is better or worse is hard to be drawn. Overall, oral administration is recommended. In addition, a subgroup analysis of the different courses of treatment was performed, and results demonstrated no significant differences in efficacy and adverse effects between the courses of treatment, echoing the results of the meta-regression, indicating that individualised treatment plans can be developed according to different patient conditions.

Compared to previous studies [12,13], this present investigation included more studies and samples, and meta-regression analysis, TSA, and Egger's test were used to verify the robustness of the results. In terms of conclusion, this study focused on immune function and compared the efficacy and safety of different Cinobufacini formulations. Reticulated meta-analyses have shown that the efficacy of Cinobufacini is comparable to that of similar Chinese medicinal preparations such as KLT, Adi, and Kang'ai for lung cancer in combination with platinum-based chemotherapy [61,62].

According to traditional Chinese medicine theory, Cinobufacini can clear heat and detoxify toxins, relieve water retention and swelling, resolve blood stasis and soften hardness [63]. Pharmacological research has found that Cinobufacini inhibits tumour cell proliferation [64,65], inducing tumour cell apoptosis [66,67], inhibiting tumour cell metastasis [68,69], reversing tumour cell chemotherapy resistance [70,71], regulating immune response [72,73]. Its potential mechanisms in lung cancer include inhibition of the AKT/mTOR signalling pathway [74] and the PI3K/AKT signalling pathway [75], regulation of the MiR-106a-5p/STAT3 signalling pathway [76], inhibition of the NF-κB/COX-2 pathway through NF-κB activation of the p65 subunit through the NF-κB/COX-2 pathway, blocking the expression of the pro-inflammatory factor COX-2 and reducing the levels of tumour necrosis factor (TNF)-κ-induced pro-inflammatory factors interleukin (IL)-6 and IL-8 messenger RNA [77], up-regulation of IL-12p70 and IL-1β secretion by dendritic cells, down-regulation of IL-10 secretion, and induction of the Th1-type cellular immune response [78]. Overall, Cinobufacini exerts adjuvant chemotherapeutic and anti-tumour effects through multiple pathways.

However, this study had some limitations. First, all included studies were conducted only in China and were flawed in terms of randomisation, allocation concealment, and blinding, which could lead to a potential risk of bias. Second, owing to the low quality of some of the included studies, several outcomes were of low quality according to the GRADE criteria. Third, there were differences in the platinum-based chemotherapy regimens used in the included studies. The dosage and duration varied among the studies when the same formulation of Cinobufacini was used. Fourth, most studies had small sample sizes, ranging from 60 to 100 in total, which reduced the reliability of the results.

## 5 Conclusion

In conclusion, compared to platinum-based chemotherapy alone, the addition of Cinobufacini can improve patient immune function, enhance efficacy, and reduce toxic side effects. This demonstrated that Cinobufacini is a safe and effective adjuvant drug for chemotherapy. In addition, the efficacies of the two mainstream formulations of Cinobufacini (i.e., capsule and injection) were comparable. However, given the shortcomings of the included studies, rigorously designed, adequately sampled, well-blinded, high-quality RCTs are anticipated to further confirm this conclusion.

## Funding information

This work was funded by the 10.13039/501100001809National Natural Science Foundation of China (No. 82074358).

## Author contribution statement

Yisheng Zhao; Dongwei Zhu: Performed the experiments; Analyzed and interpreted the data; Wrote the paper. Zhichao Wu: Analyzed and interpreted the data; Contributed reagents, materials, analysis tools or data. Dan Wang; Le Bai: Contributed reagents, materials, analysis tools or data. Xianmei Zhou: Conceived and designed the experiments.Yong Xu: Conceived and designed the experiments; Wrote the paper.

## Data availability statement

The authors do not have permission to share data.

## Declaration of competing interest

The authors declare that they have no known competing financial interests or personal relationships that could have appeared to influence the work reported in this paper.
